# The Novel Cytokine Interleukin-33 Activates Acinar Cell Proinflammatory Pathways and Induces Acute Pancreatic Inflammation in Mice

**DOI:** 10.1371/journal.pone.0056866

**Published:** 2013-02-13

**Authors:** Duraisamy Kempuraj, Erik C. Twait, Deborah E. Williard, Zuobiao Yuan, David K. Meyerholz, Isaac Samuel

**Affiliations:** 1 Department of Surgery, Roy J. and Lucille A. Carver College of Medicine, The University of Iowa, Iowa City, Iowa, United States of America; 2 Department of Pathology, Roy J. and Lucille A. Carver College of Medicine, The University of Iowa, Iowa City, Iowa, United States of America; French National Centre for Scientific Research, France

## Abstract

**Background:**

Acute pancreatitis is potentially fatal but treatment options are limited as disease pathogenesis is poorly understood. IL-33, a novel IL-1 cytokine family member, plays a role in various inflammatory conditions but its role in acute pancreatitis is not well understood. Specifically, whether pancreatic acinar cells produce IL-33 when stressed or respond to IL-33 stimulation, and whether IL-33 exacerbates acute pancreatic inflammation is unknown.

**Methods/Results:**

In duct ligation-induced acute pancreatitis in mice and rats, we found that (a) IL-33 concentration was increased in the pancreas; (b) mast cells, which secrete and also respond to IL-33, showed degranulation in the pancreas and lung; (c) plasma histamine and pancreatic substance P concentrations were increased; and (d) pancreatic and pulmonary proinflammatory cytokine concentrations were increased. In isolated mouse pancreatic acinar cells, TNF-α stimulation increased IL-33 release while IL-33 stimulation increased proinflammatory cytokine release, both involving the ERK MAP kinase pathway; the flavonoid luteolin inhibited IL-33-stimulated IL-6 and CCL2/MCP-1 release. In mice without duct ligation, exogenous IL-33 administration induced pancreatic inflammation without mast cell degranulation or jejunal inflammation; pancreatic changes included multifocal edema and perivascular infiltration by neutrophils and some macrophages. ERK MAP kinase (but not p38 or JNK) and NF-kB subunit p65 were activated in the pancreas of mice receiving exogenous IL-33, and acinar cells isolated from the pancreas of these mice showed increased spontaneous cytokine release (IL-6, CXCL2/MIP-2α). Also, IL-33 activated ERK in human pancreatic tissue.

**Significance:**

As exogenous IL-33 does not induce jejunal inflammation in the same mice in which it induces pancreatic inflammation, we have discovered a potential role for an IL-33/acinar cell axis in the recruitment of neutrophils and macrophages and the exacerbation of acute pancreatic inflammation.

**Conclusion:**

IL-33 is induced in acute pancreatitis, activates acinar cell proinflammatory pathways and exacerbates acute pancreatic inflammation.

## Introduction

Acute pancreatitis is potentially fatal when it progresses to systemic inflammation and multi-organ failure.[1] However, the mechanisms underlying the pathogenesis of acute pancreatitis are not well understood. As the elucidation of the important events in the early stages of disease progression in humans is not possible, we characterized a novel mouse model of pancreatic duct ligation-induced acute pancreatitis that is associated with systemic inflammation and substantial mortality.[2], [3] The primary objective of the present study was to examine the potential role of the novel cytokine interleukin-33 (IL-33) in the pathogenesis of acute pancreatitis. We first ascertained expression of IL-33 in our model of ligation-induced acute pancreatitis in mice. We then performed investigations to test the hypothesis that IL-33 exacerbates acute pancreatitis.

IL-33, a new member of the IL-1 superfamily of cytokines,[4] is induced in certain circumstances such as acute and chronic inflammation, cell death (“alarmin” role) and autoimmune disorders.[4]–[7] IL-33 expression is mediated via one or more of the mitogen activated protein (MAP) kinases [extracellular regulated kinase (ERK), c-Jun N-terminal kinase (JNK), p38)] and nuclear transcription factors nuclear factor-kappaB (NF-κB) and activator protein-1 (AP-1).[4]–[6] IL-33 has recently been shown to play a role in inflammatory diseases of the lung,[8], [9] joints,[10] skin,[11], [12] bowel[13] and the nervous system.[14], [15] There is accumulating evidence that IL-33 exacerbates ulcerative colitis.[6], [13], [16]–[18] There is also recent evidence that IL-33 plays a role in fibrogenesis in chronic pancreatitis.[19] However, investigations into the potential role of IL-33 in acute pancreatic inflammation are limited.[20] Specifically, whether pancreatic acinar cells respond to IL-33 or produce IL-33 in response to agonist stimulation, and whether IL-33 exacerbates the development of acute pancreatic inflammation, is not known.[19], [20] In the present study, we evaluated expression of IL-33 in pancreatic duct ligation-induced acute pancreatitis in mice and rats, isolated pancreatic acinar cell expression of and response to IL-33, and the effect of exogenous IL-33 protein on the mouse pancreas *in vivo*. Our studies have resulted in the novel findings that the IL-33 concentration is increased in the pancreas in ligation-induced acute pancreatitis, IL-33 is released from isolated pancreatic acinar cells following agonist stimulation, IL-33 activates proinflammatory pathways in pancreatic acinar cells *in vitro* and *in vivo*, and that IL-33 exacerbates acute pancreatic inflammation. We have also shown that the release of IL-33 by acinar cells and the response of acinar cells to IL-33 are mediated by ERK MAP kinase, as has been observed in several other cell types.[4], [21], [22]

To our knowledge, there has been only one report until now that examines the role of IL-33 in acute pancreatitis pathogenesis.[20] Ouziel et. al. examined choline-deficient ethionine-supplemented diet-induced acute pancreatitis and cerulein-induced acute pancreatitis in transgenic mice that are deficient in the IL-33 receptor ST2.[20] ST2-deficient mice showed increased severity of pancreatitis in both experimental models of acute pancreatitis, compared to wild type controls, suggesting that the IL-33/ST2 axis may play a *protective role* in acute pancreatitis.[20] In contrast, in the present report we show that exogenous IL-33 administered for two days induces acute inflammation in the pancreas indicating that IL-33 *exacerbates* acute pancreatitis rather than protects against it. We explain these seemingly contradictory results by suggesting that ST2-deficient mice could manifest the phenotypic effects of the absence of IL-33 influences during development, such as dysregulation of tissue healing pathways, resulting in exacerbation of tissue injury in response to an inflammatory insult. Given the dichotomous role of IL-33 in the opposing signaling pathways that either improve healing or increase the inflammatory response,[13] the finding in the present study that IL-33 exacerbates acute pancreatic inflammation is compatible with the existing literature.

As there is a documented association between IL-33 expression and mast cell activation in several inflammatory conditions,[20], [21], [23]–[26] we evaluated mast cell activation in our experimental models. We detected mast cell degranulation in the pancreas and lung in mice and rats with ligation-induced acute pancreatitis, which when taken together with increased IL-33 expression suggests that interactions between IL-33 and mast cells may play a role in disease pathogenesis. However, when we administered exogenous recombinant IL-33 for two days in mice (without duct ligation), we observed acute pancreatic inflammation in the absence of mast cell degranulation. This raises the intriguing possibility that interactions between IL-33 and the acinar cell are involved during the early stages of development of acute inflammation in the pancreas, independent of mast cell degranulation.

## Materials and Methods

### Ethics Statement

All animal experimental protocols were approved by the University of Iowa Institutional Animal Care and Use Committee (Research Protocol No. 1012246), and carried out in strict accordance with the recommendations in the Guide for the Care and Use of Laboratory Animals of the National Institutes of Health, Bethesda, MD. Human pancreas samples were obtained according to protocols approved by the University of Iowa Institutional Review Board (Permit No. 201202743; protocols involved written patient consent).

### Animal Models of Duct Ligation-Induced Acute Pancreatitis

Using aseptic precautions, and all efforts to minimize suffering, midline laparotomy was performed on mice (male C57BL/6 30–50 g, Jackson Laboratories, Bar Harbor, ME) or rats (male Sprague-Dawley 250–300 g, Harlan Sprague, Madison, WI) under general anesthesia induced and maintained with 2–5% isoflurane using a vaporizer and gas scavenger system.[2], [3] The distal bile-pancreatic duct was ligated near its junction with the duodenum to induce acute pancreatitis.[2], [3] In sham-operated controls, laparotomy was performed but the duct was not ligated. Postoperatively, the animals were carefully observed, allowed free access to food and water, and given buprenorphine analgesia (0.05 mg/kg) s.c. twice daily.[2], [3] Euthanasia was performed by decapitation under isoflurane general anesthesia at selected time points following surgery, and organs and plasma were harvested and stored at −80°C for further analysis. Portions of pancreas and lung were flash frozen in liquid nitrogen for cryostat sections or fixed in 10% neutral-buffered formalin for paraffin embedding and sectioning; additional portions of pancreas were flash frozen for other studies. Animal numbers (n) used for Enzyme-Linked Immunosorbant Assay (ELISA) for *in vivo* mouse studies are summarized in Table 1. Variations of “n” in the different ELISA measurement groups reflect availability of adequate sample to perform the stated test.

**Table 1:** Summary of animal numbers (“n”) from the *in vivo* data presented in graphs

Figure	ELISA	Sample origin	Sham (n)	PD (n)
2A: 24–72 hrs	IL-33	Mouse pancreas	3	3
2B: 48 hrs	IL-33	Rat pancreas	5	5
9A: 48 hrs	Substance P	Mouse pancreas	4	4
9A: 48 hrs	Histamine	Mouse plasma	9	9
9B: 48 hrs	Substance P	Rat pancreas	5	6
9B: 48 hrs	Histamine	Rat plasma	15	10
10A: 24 hrs	IL-1β, TNF-α	Mouse pancreas	4	4
10A: 24 & 48 hrs	IL-6	Mouse pancreas	8	8
10A: 72 hrs	IL-6	Mouse pancreas	6	5
10A: 24 hrs	IL-6	Mouse lung	4	5
10A: 48 hrs	IL-6	Mouse lung	6	6
10A: 72 hrs	IL-6	Mouse lung	3	3
10B: 24 & 48 hrs	CCL2	Mouse pancreas	6	6
10B: 72 hrs	CCL2	Mouse pancreas	6	5
10B: 24 hrs	CCL2	Mouse lung	6	6
10B: 48 hrs	CCL2	Mouse lung	6	5
10B: 72 hrs	CCL2	Mouse lung	3	3
10B: 24 hrs	CXCL2	Mouse pancreas	4	4
10B: 48 hrs	CXCL2	Mouse lung	6	4

Variations of animal number (n) in the different groups reflect availability of adequate sample. PD  =  distal bile-pancreatic duct ligation.

### Measurement of Cytokines, Chemokines, Histamine and Neuropeptide Substance P

ELISA was used to measure cytokines [IL-33, IL-1β, tumor necrosis factor-α (TNF-α), IL-6], chemokines C-C motif ligand 2 [also known as monocyte chemotactic protein-1 (CCL2/MCP-1)] and C-X-C motif ligand 2 [also known as macrophage inflammatory protein 2-alpha (CXCL2/MIP-2α)], and neuropeptide substance P concentration in pancreas and lung tissue lysates using 25–100 µg protein/sample. Tissues were homogenized in HEPES lysis buffer containing protease and phosphatase inhibitors[27] and the protein concentration was determined using the BCA assay (Pierce, Rockford, IL). TNF-α (Cat. #CMC3013) and IL-1β (Cat. #CMC0813) antibody pairs for ELISA were from Invitrogen (Camarillo, CA). IL-6 (Cat. #DY406), IL-33 (Cat. #DY3626), CCL2/MCP-1 (Cat. #DY479), CXCL2/MIP-2α (Cat. #DY452) ELISA DuoSets and substance P kits (Cat. #KGE007) were purchased from R&D Systems (Minneapolis, MN). Mouse IL-33 ELISA was used for rat samples due to high cross-reactivity (86%). Plasma histamine levels were measured with ELISA using 35–50 µl plasma/sample (Alpco Immunoassays, Salem, NH, Cat. #17-HIST-E01-RES).

### Immunoblotting

Lysates were prepared by homogenization of frozen portions of pancreas (or cell pellets) in HEPES lysis buffer.[27], [28] Samples with 40 µg total protein were denatured and subjected to SDS-PAGE on 12% gels, then transferred to PVDF membranes that were blotted against IL-33 rabbit polyclonal antibody (Santa Cruz Biotechnology, Santa Cruz, CA, Cat. #SC-98660) and goat anti-rabbit-IgG-HRP (Santa Cruz, Cat. #SC-2004). Blots were developed using ECL plus Western blotting kit and densitometry analysis was performed using Image J software. The membranes were stripped and then reprobed for β-actin (Cat. #A-5316, Sigma-Aldrich, St. Louis, MO) expression as a protein loading control. Primary antibodies against phosphorylated forms of ERK, p38, JNK and p65 (Cat. #9101, 9211, 9251 and 3033, respectively), or total ERK, p38, JNK and p65 (Cat #9102, 9212, 9252, and 4764, respectively) were purchased from Cell Signaling Technology, Inc. (Danvers, MA) and used for immunoblotting where indicated.

### Pancreatic IL-33 mRNA Real Time-Polymerase Chain Reaction (RT-PCR)

Total RNA was isolated from flash frozen pancreas of sham or pancreatic duct ligated mice at 3, 5, or 24 hrs using RNeasy Mini Kit (Qiagen, Valencia, CA) and then converted to cDNA using iScript cDNA Synthesis Kit (Bio-Rad, Hercules, CA). TaqMan probes (Applied Biosystems, Foster City, CA) specific for mouse IL-33 (TaqMan assay I.D. Mm00505403_m1), and for 18S rRNA (TaqMan assay I.D. Mm03928990_g1), were used in real time amplifications using a CFX96 system (Bio-Rad). IL-33 mRNA levels were normalized to 18S controls and quantified by the ΔΔCt method.

### IL-33 Immunohistochemistry (IHC) in Pancreas and Lung

IL-33 IHC was carried out as per kit instructions on 6 µm cryostat sections of rodent pancreas and lung using IL-33 rabbit polyclonal primary antibody (1∶200 dilutions, Santa Cruz, Cat. #SC-98660), and ultra-sensitive ImmPRESS reagent anti-Rabbit Ig peroxidase (Cat. #MP-7401) and ImmPACT DAB peroxidase substrate (Cat. #SK-4105), both from Vector Laboratories (Burlingame, CA). Development of brown color indicated a positive reaction for IL-33. In negative controls the primary antibody was excluded.

### Isolated Pancreatic Acinar Cell Preparation

Pancreatic acinar cells were isolated from mice using a collagenase (Worthington Biochemical, Lake Wood, NJ) digestion procedure.[28], [29] Cells were maintained at 37°C in 95% air and 5% CO_2_. Cells were seeded in 12-well culture plates (350–400 µg protein/well) using Dulbecco's Modified Eagle Medium (Life Technologies, Grand Island, NY, Cat. #11965) containing 5% fetal bovine serum and 1% penicillin-streptomycin. Following overnight equilibration, cells were stimulated with mouse recombinant IL-33 with or without luteolin (Cayman Chemical, Ann Arbor, MI, Cat. #10004161) pretreatment for 15 min. The culture medium was assayed for IL-6 and CCL2/MCP-1 release by ELISA. In separate studies, the isolated acinar cells were incubated with adenovirus expressing dominant negative ERK (Ad.DN.ERK2) for 48 hrs prior to stimulation, with controls incubated with empty virus (Ad.EV) or adenovirus expressing green fluorescent protein (Ad.GFP), using a multiplicity of infection (MOI) of 5. Replication-deficient recombinant adenoviruses expressing DN.ERK2 (dual phosphorylation site T202/Y204 mutated to A202/F204) or GFP, or not expressing a transgene, were made by the University of Iowa Vector Core Facility (Iowa City, IA).

We have previously established our isolated acinar cell preparation and culture technique with good cell viability shown by adenosine triphosphate (ATP) assay and strong cell response to cholecystokinin stimulation measured by amylase release, while GFP expression visualized by fluorescent microscopy confirmed nearly 100% infection efficiency.[28] In the present study, we also prepared acinar cell smears for morphological analysis, at time zero and after 24 hrs in culture, using a cytocentrifuge (Shandon Inc., Pittsburgh, PA). The cell smears were fixed with acetone and methanol prior to staining with 0.1% toluidine blue and showed intact cell membranes and acinar cell morphology (data not shown), confirming previous observations.[28]

### Exogenous IL-33 Protein Administration in Mice

Recombinant mouse IL-33_109-266_ protein (rIL-33)(Cat. #ALX-522-101-C10) was purchased from Enzo Life Sciences, Inc. (Farmingdale, NY). We injected five mice with rIL-33 protein [2.5 µg in 100 µL phosphate buffered saline (PBS)] i.p. two times a day for two days and the mice were euthanized 48 hrs following the initial injection. Others have used 4 µg IL-33 i.p. once daily for 7 days to elicit morphological changes in the jejunum, spleen and lung.[4], [30] Instead, we chose to examine the 48-hour time point in our study based on the reasoning that acinar cell responses to IL-33 could induce morphological changes in the pancreas much earlier than in other organs. Mice injected with an equal volume of PBS i.p. were used as controls (n = 3). The pancreas was excised and one portion was frozen in liquid nitrogen for immunoblotting, a second portion was fixed in 10% neutral-buffered formalin for morphological studies and the rest was processed immediately to prepare isolated acinar cells for measurement of IL-6 and CXCL2/MIP-2α release into the medium using ELISA. The lung and jejunum were also excised and fixed for morphological studies.

### Evaluation of Pancreatic and Pulmonary Mast Cell Activation

To evaluate mast cell activation, known to be associated with IL-33 expression, we stained sections of pancreas and lung with 0.1% toluidine blue (Sigma, Cat. #89640). Histological evidence of mast cell activation was elucidated by detecting the presence of extracellular granules (degranulation) associated with reduced mast cell staining.

### Human Pancreatic Fragment Preparation

A fresh human pancreas specimen was obtained from the normal appearing resection margin of a distal pancreatectomy specimen from a 39-year-old female with episodes of acute on chronic pancreatitis, where the preoperative computerized axial tomogram scan showed only atrophy but no calcifications, cystic lesions or ductal dilation. The fresh portion of pancreas was quickly sliced into small fragments in culture medium, and equilibrated for 2 hrs in an incubator prior to stimulation with 100 ng/ml IL-33 for 6 hrs, using unstimulated controls for comparison (maintenance conditions as for isolated acinar cell preparations). The fragments were then harvested and subjected to immunoblotting using primary antibody to phosphorylated ERK, and total ERK immunoblots were prepared by stripping and reprobing the membranes.

### Statistical Analysis

Statistical computations were performed using InStat 3 (GraphPad Software, Inc., La Jolla, CA). All data are presented as mean ± SEM. Differences between multiple experimental groups were analyzed using one-way Analysis of Variance (ANOVA). Tukey-Kramer analysis was performed to evaluate significant differences between groups. Student's t-test was used when comparing only two conditions. A p-value less than 0.05 was considered statistically significant.

## Results

**Figure 1:**
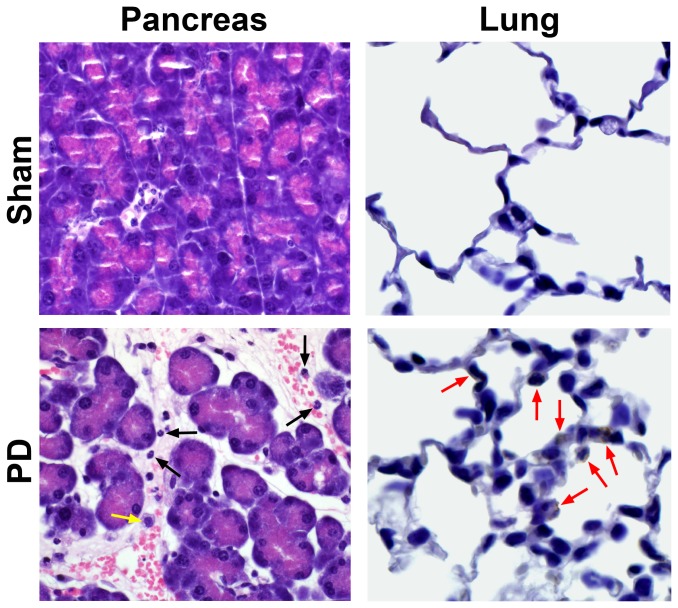
Distal bile-pancreatic duct ligation in the mouse results in acute inflammatory changes in the pancreas and lung. Representative photomicrographs (original magnification X600) of mouse pancreas and lung of sham operated controls and pancreatic duct ligated groups at 48-hr time points are shown. The pancreas from the diseased group shows interacinar and interlobular edema, vascular congestion with neutrophils, and interstitial infiltration by neutrophils and the occasional macrophage (H & E stain, black arrows: neutrophils, yellow arrow: macrophage). The lung from the diseased group shows thickening of alveolar septa associated with increased neutrophils (red arrows, MPO immunohistochemistry).

Morphological studies of pancreas and lung from mice with 48 hrs of ligation-induced acute pancreatitis showed changes confirming development of acute pancreatitis and acute lung injury (Fig. 1), as reported previously in greater detail.[2], [3] ELISA of TNF-α and IL-1β in bronchoalveolar lavage fluid and plasma showing evidence of systemic inflammation, and immunoblots showing pancreatic ERK activation in these mice have been reported previously.[3] In the present report we focus mainly on the investigation of IL-33 levels and mast cell activation using the stored samples obtained from these mice, and we extend our studies to evaluate cytokine levels in the pancreas and lung.

### Pancreatic Duct Ligation Increases Pancreatic IL-33 Expression

**Figure 2:**
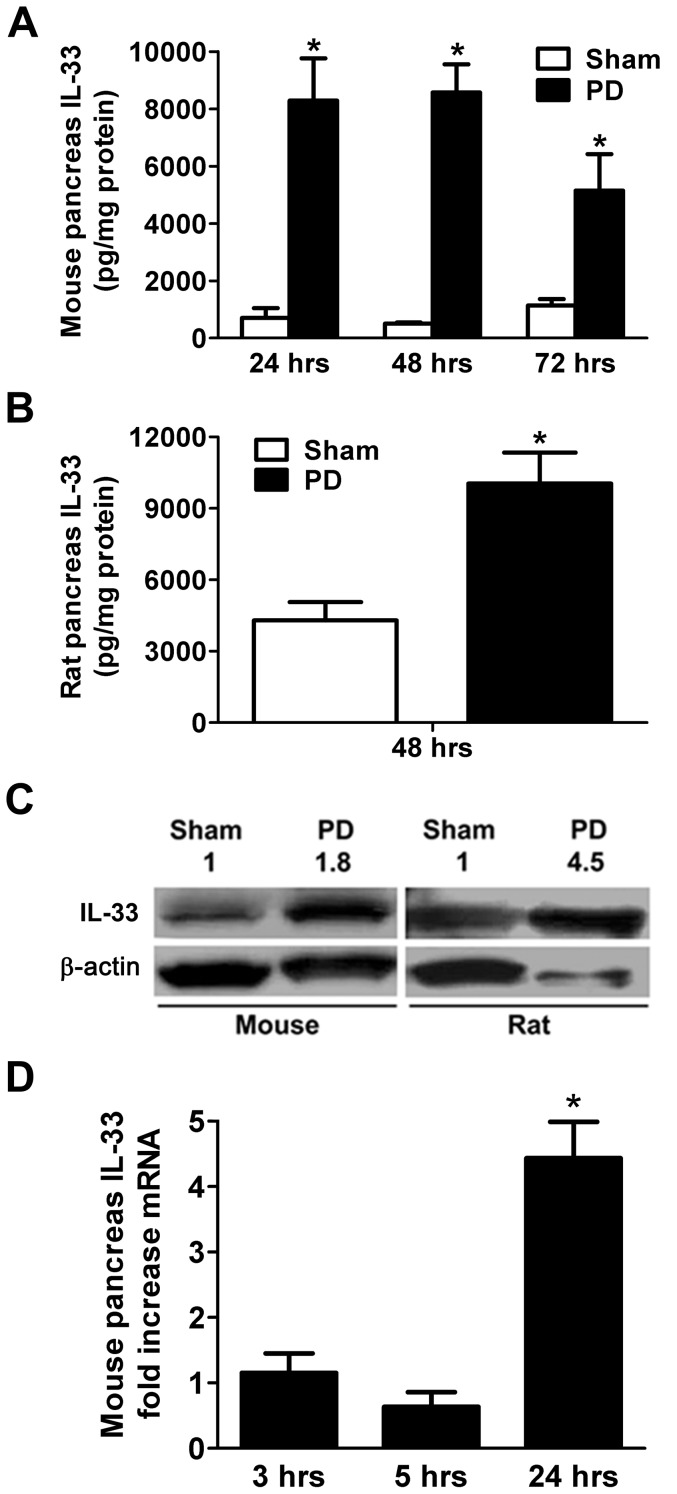
Increased pancreatic IL-33 following ligation-induced acute pancreatitis. A & B) ELISA analysis showed increased IL-33 protein concentration following pancreatic duct ligation (PD) in comparison to the sham group in: A) mouse pancreas homogenates at 24, 48 and 72 hrs, and B) rat pancreas homogenates at 48 hrs. Data are mean ± SEM; Student's t-test; asterisk (*) indicates significance compared to sham group of the corresponding time point, p<0.05. C) Immunoblots of pancreas homogenates showed increased IL-33 protein expression 48 hrs after PD compared to sham group in mice and rats. Densitometric ratios of IL-33 to β-actin expression were normalized to sham controls. D) Pancreata of mice 3, 5, or 24 hrs following sham or PD were subjected to real-time PCR analysis. The pancreatic IL-33 mRNA expression level after 24 hrs of PD relative to sham controls was increased compared to 3 or 5 hrs of PD. Fold change values are shown as mean ± SEM, n = 3/time point/condition. Asterisk (*) indicates significance over 3 and 5 hrs of PD; ANOVA; p<0.001.

Samuel et al were the first to develop a fatal mouse model of distal common bile-pancreatic duct ligation-induced acute pancreatitis (PD), a model characterized by systemic inflammation and multiorgan dysfunction.[2] We investigated IL-33 protein concentration in the pancreas in this mouse model using ELISA. We observed a several-fold increase in pancreatic IL-33 concentration in mice after 24 hrs of PD and this increase was also apparent at 48 and 72 hrs (Fig. 2A).

Encouraged by our findings in the mouse model, we extended our studies to the non-fatal rat model of pancreatic duct ligation-induced acute pancreatitis.[31], [32] Using ELISA, we detected a greater than 2-fold increase in IL-33 protein concentration in the rat pancreas after 48 hrs of PD, compared to sham controls (Fig. 2B). Increased expression of IL-33 protein in pancreata of mice and rats after PD was corroborated with immunoblotting (Fig. 2C). Using RT-PCR (n = 3 mice per time point per condition), increased expression of IL-33 mRNA in the mouse pancreas was confirmed after 24 hrs of PD (relative to 24-hour sham controls) and was significantly higher than after 3 or 5 hrs of PD (relative to 3- and 5-hour sham controls, respectively)(Fig. 2D). IL-33 mRNA expression after 3 or 5 hrs of PD was not different from corresponding sham control values.

### Pancreatic Duct Ligation Increases IL-33 Within Infiltrating Cells in Pancreas and Lung

**Figure 3:**
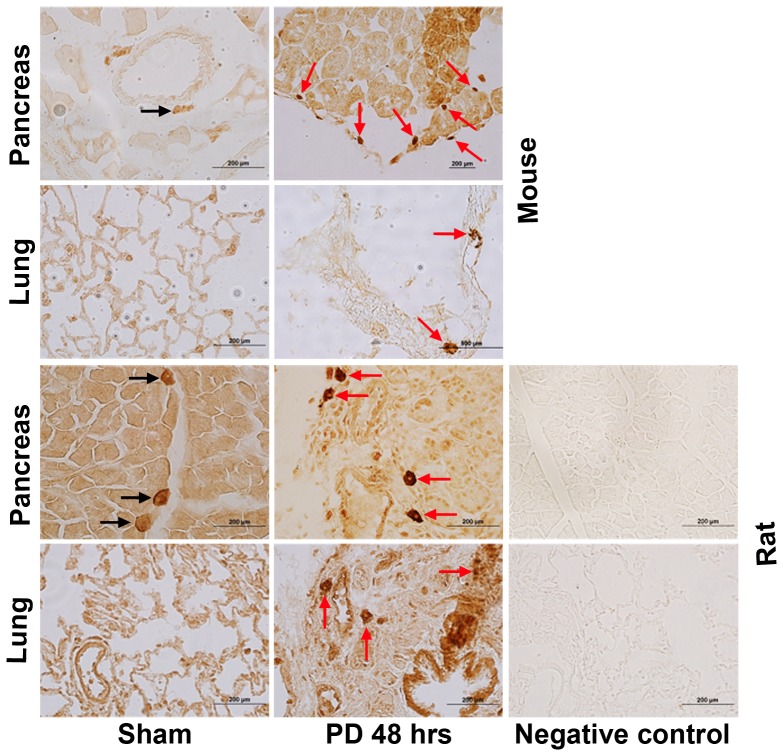
Increased IL-33 in infiltrating cells in the pancreas and lung in ligation-induced acute pancreatitis. Immunohistochemistry using pancreas and lung tissue cryosections showed increased IL-33 staining of infiltrating cells 48 hrs after PD compared to sham control group in mice and rats. Small compact cells that stain intensely for IL-33 are most probably mast cells. Black arrows show IL-33 stained infiltrating cells in sham controls while red arrows show IL-33 stained infiltrating cells with increased IL-33 staining 48 hrs after PD. Brown color indicates positive IL-33 reaction. Negative controls lack primary antibody. Scale bar  = 200 µm.

Immunohistochemistry using specific antibody to IL-33 showed increased staining of infiltrating cells within the pancreas and lung after 48 hrs of PD in mice and rats (Fig. 3). These infiltrating cells that stain intensely for IL-33 are most probably mast cells. Although neutrophils and eosinophils are also other possibilities, mast cells are typically known to produce abundant IL-33 when activated and thus would be expected to stain with this intensity.[33] The corresponding staining did not occur in negative control experiments (in which the primary antibody was omitted) in the pancreas and lung of rats after 48 hrs of PD, and was not as intense in sham-operated controls. Constitutive expression of IL-33 in the pancreas is also evident and has been reported in detail by two different groups.[19], [20]

### TNF-α Stimulation Increases IL-33 Release from Pancreatic Acinar Cells

**Figure 4:**
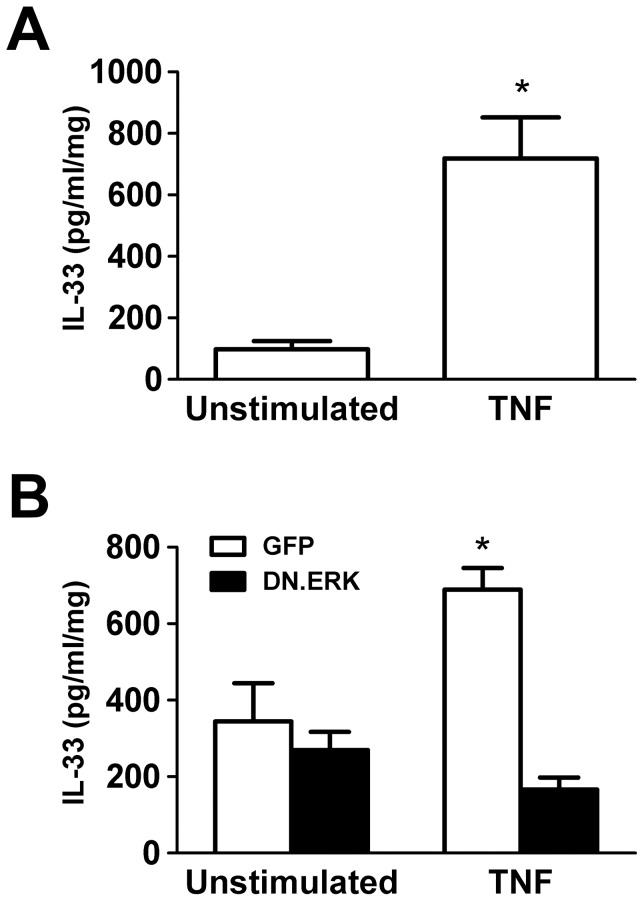
TNF-α stimulates IL-33 release from acinar cells, involving the ERK MAP kinase pathway. A) Isolated mouse acinar cells stimulated with TNF-α (10 ng/ml) for 6 hrs showed increased IL-33 release into the medium. B) Isolated mouse acinar cells were infected with 5 MOI of Ad.GFP or Ad.DN.ERK for 48 hrs and then stimulated with TNF-α (10 ng/ml) for 6 hrs before collecting medium for ELISA analysis. IL-33 levels in the medium were normalized to total protein in cells. Data are mean ± SEM; n = 3 wells/group; ANOVA, asterisk (*) indicates significance compared to unstimulated control, p<0.05.

Whether or not pancreatic acinar cells increase IL-33 release when stressed is not known.[19], [20] Isolated mouse acinar cells stimulated with TNF-α (10 ng/ml) for 6 hrs showed a 7-fold increase in IL-33 release into the medium (ELISA), compared to unstimulated controls (Fig. 4A). We also evaluated whether specific inhibition of ERK diminishes TNF-α-stimulated IL-33 release. We found that 48 hrs of pre-incubation with 5 MOI Ad.DN.ERK2 abrogated TNF-α-stimulated (10 ng/ml for 6 hrs) increases in acinar cell IL-33 release to levels not higher than unstimulated controls pre-incubated with 5 MOI Ad.GFP for 48 hrs (Fig. 4B). These findings indicate that mouse pancreatic acinar cells increase IL-33 release in response to TNF-α stimulation via the ERK MAP kinase pathway.

### Mouse Isolated Acinar Cells Respond to IL-33

Several cell types respond to IL-33 stimulation with production of inflammatory mediators, involving MAP kinases such as ERK and also nuclear transcription factors NF-κB and AP-1.[4] However, the response of pancreatic acinar cells to stimulation by IL-33 is not known.[19], [20]

**Figure 5:**
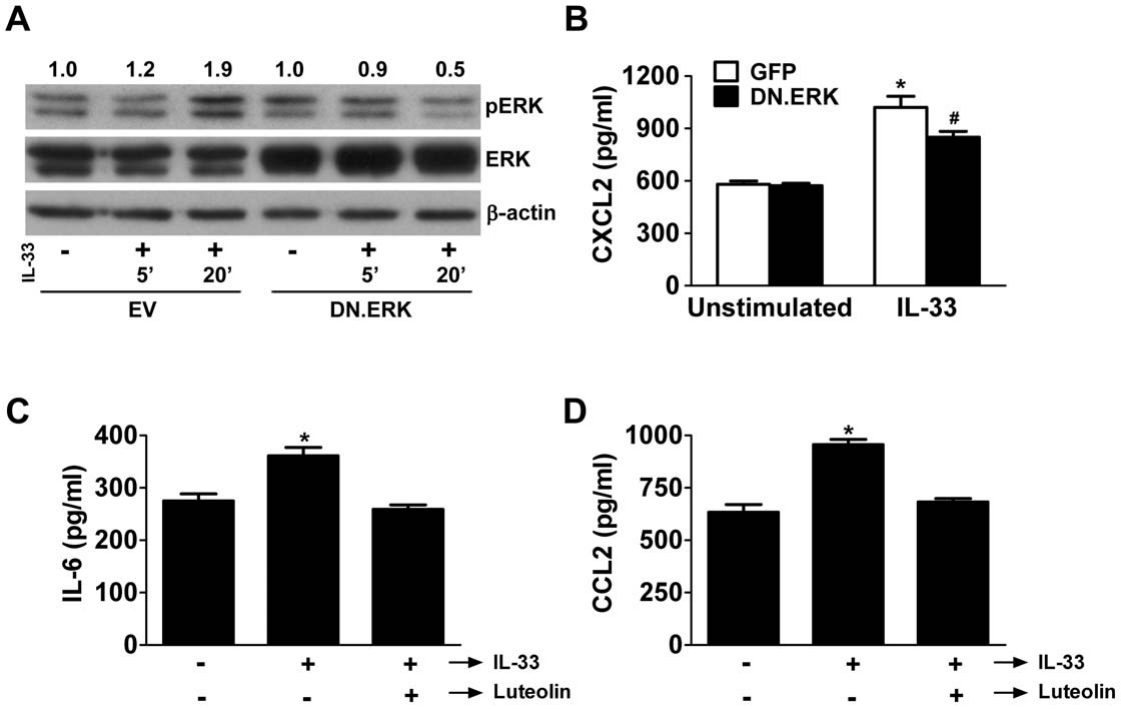
IL-33 stimulates ERK activation and cytokine release from mouse acinar cells. A) ERK is activated in mouse acinar cells following IL-33 stimulation, and this activation is inhibited by DN.ERK expression. Cells were infected with 5 MOI of Ad.EV or Ad.DN.ERK2 for 48 hrs and then stimulated with IL-33 (100 ng/ml) for 5 or 20 min. β-Actin expression was used as a protein loading control, while increased ERK expression verified overexpression of DN.ERK. Densitometric ratios of pERK to β-actin expression were normalized to unstimulated controls. B) IL-33 stimulation leads to increased CXCL2/MIP-2α release from mouse acinar cells which is inhibited by DN.ERK expression. Cells were infected with 5 MOI of Ad.GFP or Ad.DN.ERK2 for 48 hrs and then stimulated with IL-33 (100 ng/ml) for 6 hrs before collecting medium for ELISA analysis. CXCL2/MIP-2α levels in the medium were normalized to total protein in cells. Data are mean ± SEM; n = 3 wells/group; ANOVA, asterisk (*) indicates significance compared to unstimulated control, pound sign (#) indicates significance versus stimulated control and unstimulated control, p<0.05. (C & D) IL-33 stimulation of mouse acinar cells leads to increased: C) IL-6 release and D) CCL2/MCP-1 release. Cells were stimulated with IL-33 (25 ng/ml) for 6 hrs before collecting medium for ELISA analysis. Pretreatment with luteolin (10 µM) for 15 min inhibited both IL-6 and CCL2/MCP-1 release compared to untreated cells following stimulation. Data are mean ± SEM; n = 3 wells/group; ANOVA; asterisk (*) indicates significance compared to unstimulated control, p<0.05.

To evaluate acinar cell responses to IL-33, we first looked at the effect of IL-33 stimulation on ERK activation. We stimulated acinar cells with 100 ng/ml IL-33 after pre-incubation for 48 hrs with 5 MOI of either EV or Ad.DN.ERK2. In cells pre-incubated with EV, immunoblots showed that IL-33 stimulation was associated with increased activation of ERK, compared to unstimulated controls (20% and 90% increase after 5 and 20 min, respectively, using densitometric analysis with correction for actin expression)(Fig. 5A), indicating that IL-33 activates ERK; in cells pre-incubated with Ad.DN.ERK2, expression of the DN.ERK2 transgene was reflected by increased ERK expression and by abrogation of IL-33-induced ERK activation.

Next, we investigated whether IL-33 stimulation would cause chemokine release from acinar cells, and if so, whether specific inhibition of ERK would dampen the release. Isolated acinar cells were stimulated for 6 hrs with 100 ng/ml IL-33 after pre-incubation for 48 hrs with 5 MOI of either Ad.GFP or Ad.DN.ERK2, and we measured CXCL2/MIP-2α release into the medium with ELISA. Stimulation with IL-33 increased acinar cell CXCL2/MIP-2α release in Ad.GFP infected cells and this increase was limited in Ad.DN.ERK2 infected cells (Fig. 5B). This provides further evidence that mouse pancreatic acinar cells respond to IL-33 and that the ERK MAP kinase is involved in this signaling pathway.

Additionally, we stimulated isolated acinar cells with 25 ng/ml IL-33 for 6 hrs and measured the release of IL-6 and CCL2/MCP-1 into the medium using ELISA. We also evaluated the effect of luteolin, a commonly used flavonoid with wide-ranging anti-inflammatory properties,[34] in inhibiting acinar cell responses to IL-33 (Fig. 5C & D). Luteolin inhibits the ERK MAP kinase pathway in certain cell types.[34] Stimulation with IL-33 increased acinar cell release of IL-6 and CCL2/MCP-1, confirming that acinar cells respond to IL-33 with inflammatory mediator production. Pretreatment with 10 µM luteolin abrogated IL-33-induced acinar cell production of IL-6 and CCL2/MCP-1.

### Exogenous IL-33 Induces Acute Pancreatic Inflammation in Mice

IL-33 potentially has a dichotomous role that could either entail promotion of homeostasis with healing of injured tissue or exacerbation of inflammation.[13] We hypothesize that IL-33 exacerbates acute pancreatic inflammation. To test this hypothesis, we evaluated the effect of exogenous IL-33 protein on the mouse pancreas *in vivo*. Mice administered rIL-33 intraperitoneally were compared to controls that received PBS vehicle only.

**Figure 6:**
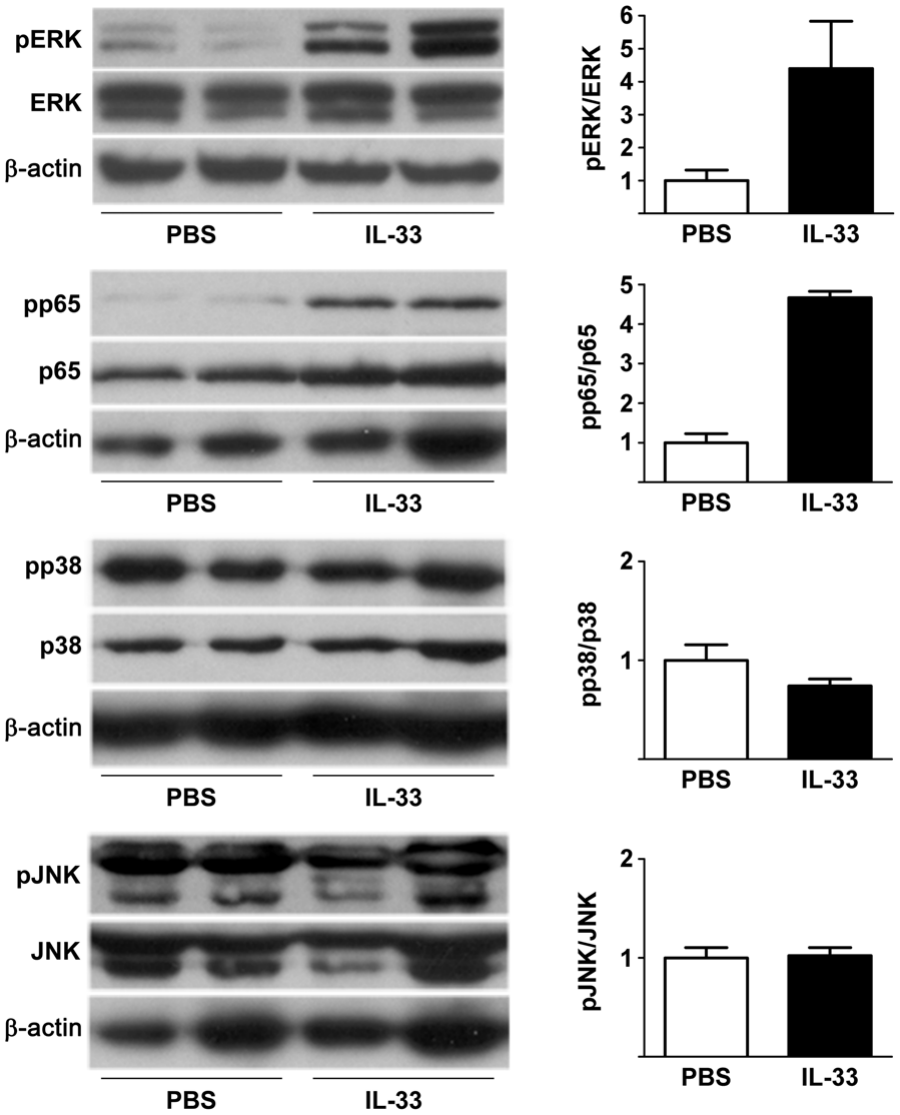
Exogenous IL-33 activates ERK and NF-κB p65 subunit in mouse pancreas. In mice (without duct ligation) injected with 2.5 µg recombinant IL-33 protein i.p. twice daily for 48 hrs, immunoblots of pancreatic homogenates showed activation of ERK and the p65 NF-κB subunit as evidenced by increased phosphorylation of ERK and p65, compared to phosphate buffered saline (PBS) injected controls, while p38 and JNK did not show evidence of activation. Densitometric ratios of phosphorylated to total protein expression were normalized to PBS controls and are graphed on the right (mean ± SEM of the two samples in each group is shown).

Immunoblotting of pancreas homogenates showed activation of ERK in rIL-33 injected mice, as evidenced by a 4.4-fold increase in phospho-ERK expression (Fig. 6). In rIL-33 injected mice, there was also a 4.7-fold increase in pancreatic expression of the phosphorylated form of the NF-κB subunit p65, while p38 and JNK did not show increased activation.

**Figure 7:**
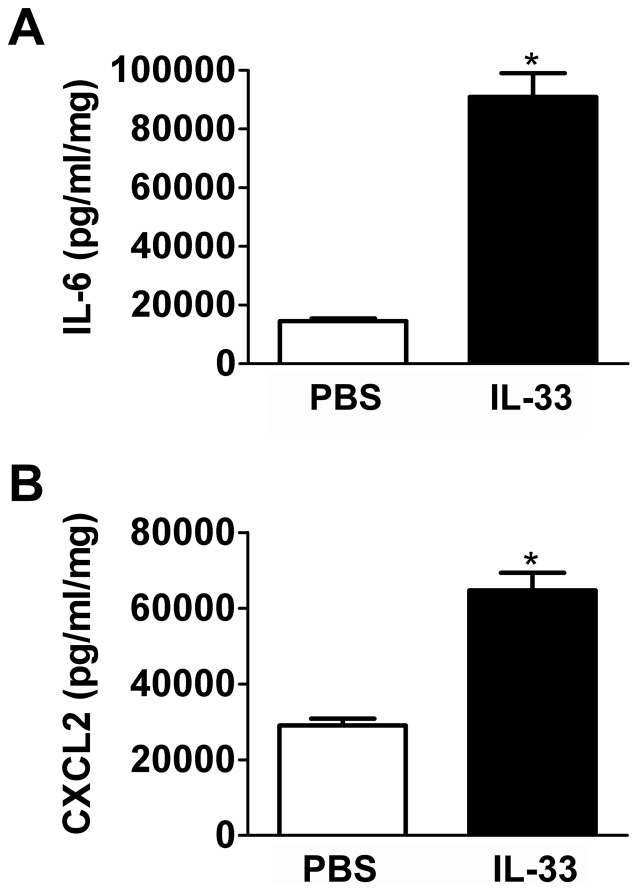
Acinar cells from mice receiving exogenous IL-33 show increased cytokine release. In mice (without duct ligation) injected with 2.5 µM recombinant IL-33 protein i.p. twice daily for 48 hrs followed by acinar cell isolation and incubation in culture medium for 6 hrs, IL-6 release and CXCL2/MIP-2α release were significantly increased compared to PBS injected controls. Both IL-6 and CXCL2/MIP-2α levels were normalized to total protein in cells. Data are mean ± SEM; n = 6 wells/group; Student's t-test; asterisk (*) indicates significance compared to PBS injected control, p<0.05.

After a 2-hour equilibration period in culture medium, isolated acinar cells from pancreata of these mice were incubated in fresh culture medium for 6 hrs to evaluate spontaneous cytokine release. ELISA of the medium showed increased release of IL-6 and CXCL2/MIP-2α from acinar cells isolated from rIL-33 injected mice, compared to PBS injected controls (Fig. 7).

**Figure 8:**
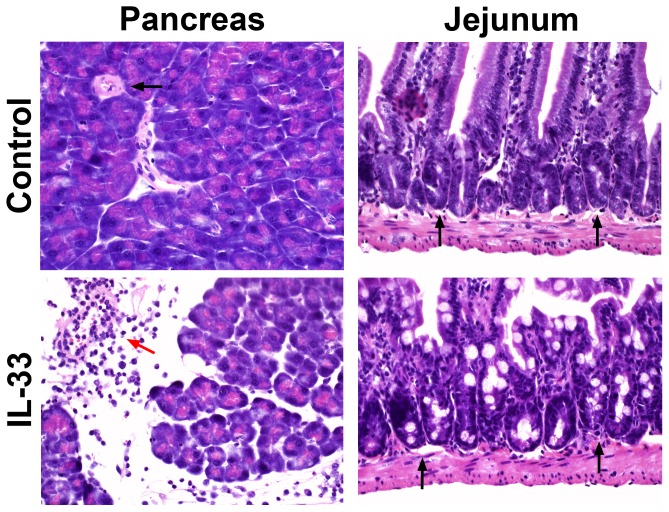
Exogenous IL-33 induces acute inflammation in the pancreas but not the jejunum. Phosphate buffered saline (PBS) injected control mice lacked pancreatic or jejunal inflammatory changes. In mice (without duct ligation) injected with 2.5 µg recombinant IL-33 protein i.p. twice daily for 48 hrs, histological examination of pancreas tissue showed multifocal expansion of perilobular areas with perivascular edema, neutrophils and some macrophages, with the consistent finding of margination and perivascular neutrophil infiltration (red arrow). Jejunum of IL-33 injected mice showed increased goblet cell density but no evidence of perivascular neutrophil infiltration (black arrows) or acute inflammation. Hematoxylin and eosin stain; original magnification X400.

Hematoxylin- and eosin-stained sections of paraffin-embedded portions of pancreas showed multifocal perivascular inflammation and interlobular edema in mice that received rIL-33 but not in the control group that received vehicle only (Fig. 8). The most consistent morphological finding in the pancreas of rIL-33 injected mice was the presence of perivascular white blood cell (WBC) infiltration consisting predominantly of neutrophils and some macrophages, with evident margination of neutrophils. As IL-33 has an established association with mast cell activation,[21] we stained new pancreatic sections with toluidine blue but failed to detect increased mast cell numbers or degranulation in rIL-33-injected mice compared to PBS-injected controls (data not shown); however, it is important to note that IL-33 is known to induce release of chemoattractants from mast cells without degranulation.[20], [21], [35] The lungs from IL-33-injected mice did not show acute inflammatory changes compared to controls (data not shown).

As intraperitoneally administered IL-33 at similar doses is not known to induce perivascular WBC infiltration in the jejunum even after seven days of daily injection,[4], [30] we compared the pancreatic morphological changes to those in the jejunum resected from the same mice (Fig. 8). Consistent with the findings of other investigators,[4], [30] jejunal sections from rIL-33-injected mice showed increased goblet cell density but *did not show* overt inflammatory changes, as seen in the pancreas from the same mice. These observations suggest that the morphological differences seen between the pancreas and jejunum in response to exogenous IL-33 administration are most probably due to IL-33-induced activation of acinar cell inflammatory pathways and consequent release of acute inflammatory mediators from acinar cells.

### Mast Cell Activation in Ligation-Induced Acute Pancreatitis

**Figure 9:**
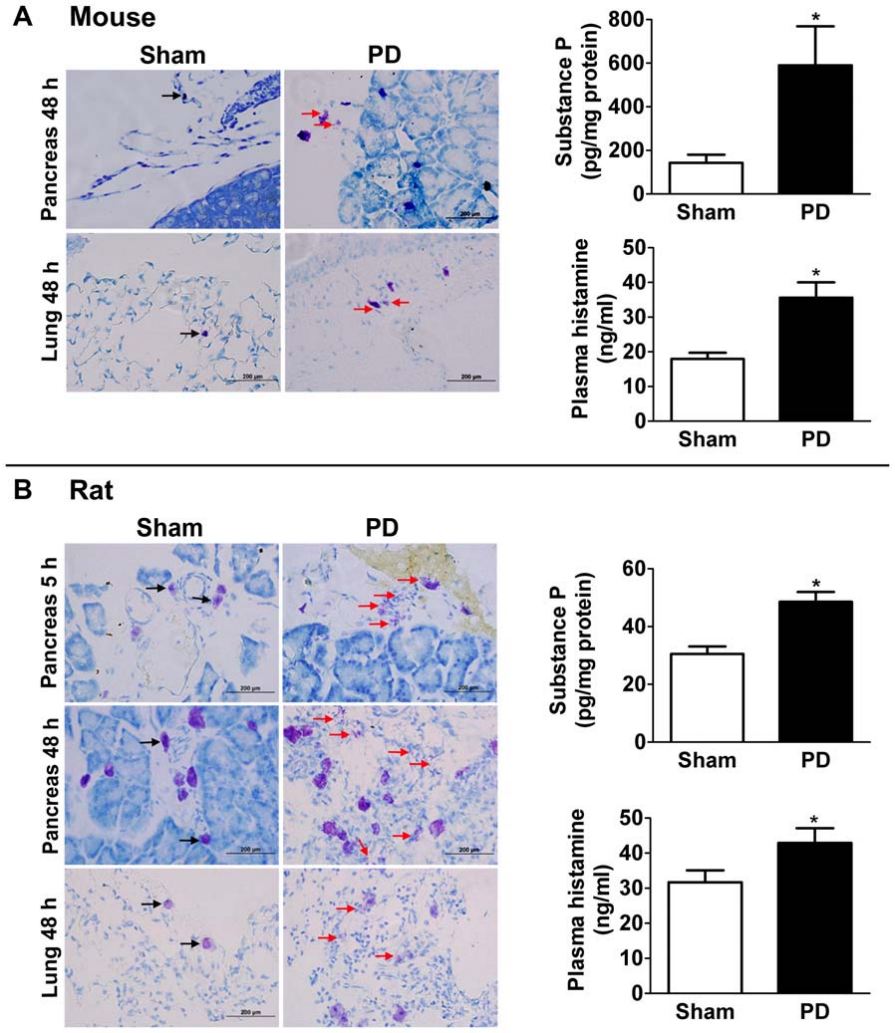
Mast cells are activated, and substance P and histamine levels are increased, in ligation-induced acute pancreatitis in mice and rats. A) Toluidine blue (0.1%) staining of mouse pancreas and lung tissue cryosections (scale bar = 200 µm) showed increased mast cell activation 48 hrs after pancreatic duct ligation (PD). Black arrows show quiescent mast cells while red arrows indicate mast cell activation (degranulation). ELISA analysis showed increased substance P in mouse pancreas homogenates and increased histamine in mouse plasma 48 hrs after PD compared to sham control group. B) Toluidine blue (0.1%) staining of rat pancreas and lung tissue cryosections (scale bar = 200 µm) showed increased mast cell activation 48 hrs after PD (also at 5 hrs in the pancreas). Black arrows show quiescent mast cells while red arrows indicate mast cell activation (degranulation). ELISA showed increased pancreatic substance P and plasma histamine in rats 48 hrs after PD. Data are mean ± SEM; Student's t-test; asterisk (*) indicates significance compared to sham control group, p<0.05.

We have shown that IL-33 is induced in ligation-induced acute pancreatitis in mice and rats (Fig. 2). Others have shown that IL-33 activates mast cells[21] and that mast cells express IL-33.[4] Several reports have implicated a role for mast cells in exacerbating acute pancreatitis or acute lung injury.[26], [34], [36]–[39] Therefore, we investigated mast cell activation in the pancreas and lung of mice and rats with ligation-induced acute pancreatitis. Toluidine blue-stained cryosections of pancreas and lung showed evidence of mast cell activation (degranulation) in pancreatitic mice and rats at the 48-hour time point that was not seen in sham-operated controls (Fig. 9A & B). Mast cell degranulation was seen as early as 5 hrs after duct ligation in the rat pancreas (Fig. 9B).

As histamine is produced predominantly by mast cells and its release is indirect evidence of mast cell activation,[40] we measured its plasma concentration using ELISA; we detected increased histamine levels in the plasma of pancreatitic mice and rats (Fig. 9).

Substance P is a neuropeptide secreted by neural cells, and also by pancreatic acinar cells,[41] that mediates inflammation partly via mast cell activation, and IL-33 augments effects of substance P on mast cells.[12] Therefore, we measured substance P in pancreas homogenates from mice and rats and found increased levels following 48 hrs of pancreatic duct ligation (ELISA, Fig. 9).

### Cytokine Production in Ligation-Induced Acute Pancreatitis in Mice

**Figure 10:**
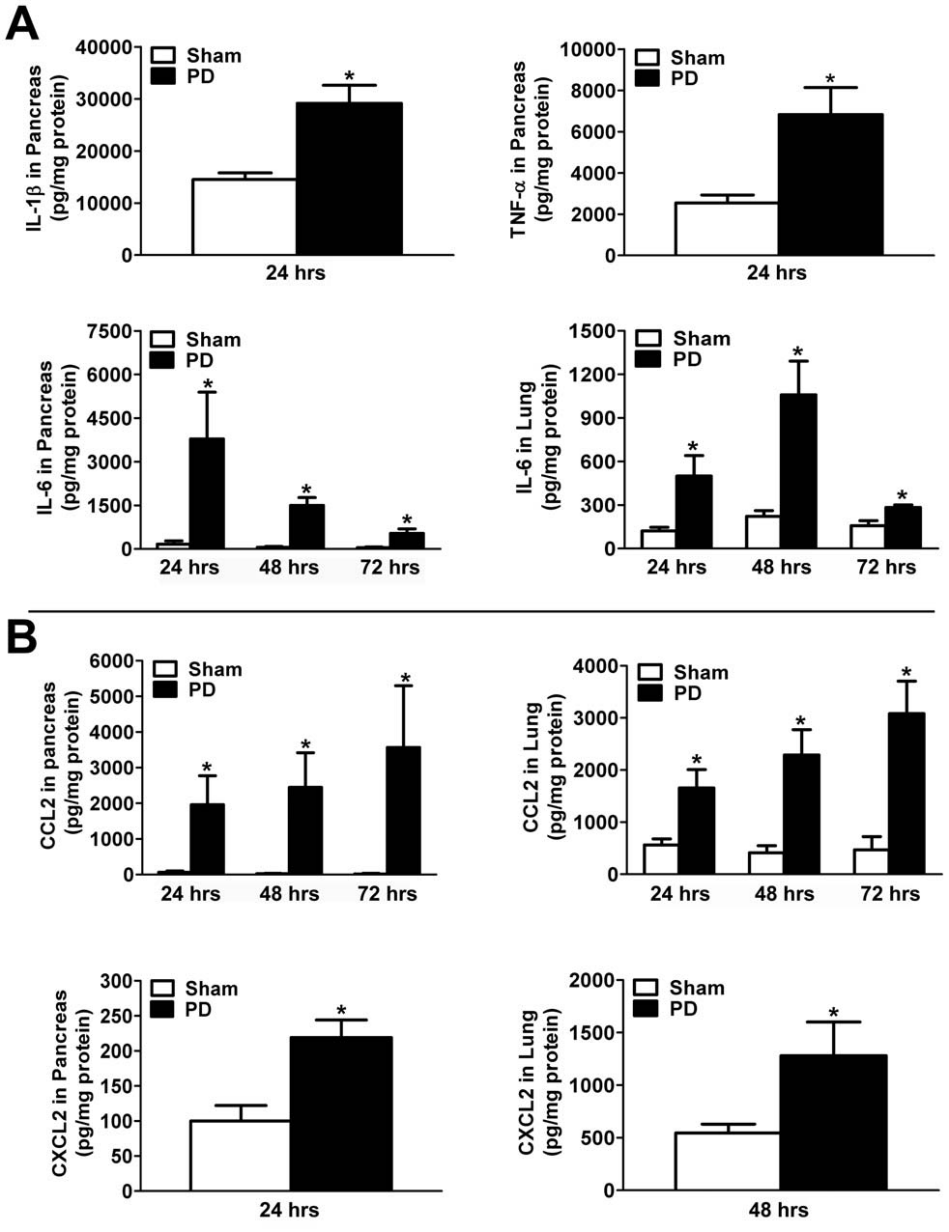
Cytokine and chemokine levels are increased in the mouse pancreas and lung in ligation-induced acute pancreatitis. A) *Cytokines* IL-1β, TNF-α and IL-6 were assayed in the pancreas of mice after 24, 48 or 72 hrs of pancreatic duct ligation (PD)(IL-6 was also assayed in the lung). B) *Chemokines* CCL2/MCP-1 and CXCL2/MIP-2α were assayed in the pancreas and lung of mice after 24, 48 or 72 hrs of PD. There was a significant increase in cytokine and chemokine concentration in the tissue homogenates from pancreatitic mice versus sham control groups. ELISA, data are mean ± SEM; Student's t-test; asterisk (*) indicates significance versus sham control group at the corresponding time point, p<0.05.

IL-1β and TNF-α are key cytokines implicated in the initiation and propagation of acute pancreatic inflammation in various experimental models of acute pancreatitis.[42], [43] Using ELISA, we confirmed increased IL-1β and TNF-α concentration in the pancreas of mice within 24 hrs of ligation-induced acute pancreatitis (Fig. 10).

To correlate our *in vitro* findings of increased acinar cell production of IL-6, CXCL2/MIP-2α and CCL2/MCP-1 in response to IL-33 stimulation with their expression *in vivo* in our experimental model, we assayed these cytokines in the pancreas and lung in ligation-induced acute pancreatitis in mice using ELISA. We found increased concentration of IL-6, CXCL2/MIP-2α and CCL2/MCP-1 in the pancreas and lung of pancreatitic mice (Fig. 10)(most levels were increased as early as 24 hrs after PD; CXCL2/MIP-2α in the lung was increased after 48 hrs of PD).

### IL-33 Activates ERK in Human Pancreatic Fragments

**Figure 11:**
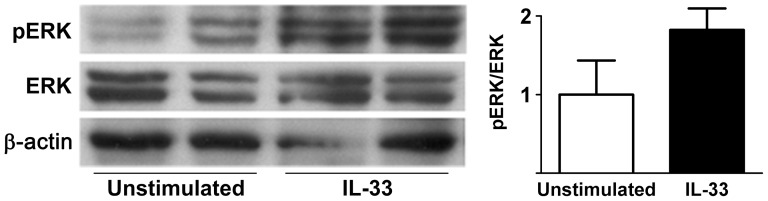
IL-33 activates ERK in the human exocrine pancreas. Human normal pancreas fragments were incubated in culture medium and stimulated with 100 ng/ml IL-33 for 6 hrs. The fragments were then processed for immunoblotting using specific antibodies to pERK, then stripped and reprobed for ERK and β-actin expression. Densitometric ratios of pERK to ERK expression were normalized to unstimulated controls (mean ± SEM of the two samples in each group is shown). IL-33 activated ERK in human pancreatic tissue as evidenced by a nearly two-fold increase in pERK expression.

The ability of IL-33 to activate inflammatory pathways in human pancreatic tissue would have important clinical implications for several of the novel findings reported here. Therefore, we evaluated whether IL-33 activates ERK in human pancreatic fragments and found a nearly two-fold increase in phosphorylation of ERK following IL-33 stimulation (Fig. 11).

## Discussion

The most important and unique aspect of the present report is the new evidence presented that the novel cytokine IL-33 exacerbates acute pancreatic inflammation. Furthermore, we demonstrate that pancreatic acinar cells increase IL-33 release following stimulation and that acinar cells release acute inflammatory mediators in response to IL-33 stimulation. Taken together with our finding of increased IL-33 concentration in the pancreas in ligation-induced acute pancreatitis, we have demonstrated a novel role for IL-33 in the exacerbation of acute pancreatic inflammation. The confirmation by other investigators that IL-33 is expressed by human pancreatic acinar cells and that the IL-33 receptor ST2 is increased in the circulation of acute pancreatitis patients emphasizes the potential clinical relevance of our findings. We have taken the potential clinical relevance one step further by demonstrating ERK activation in human pancreatic fragments in response to IL-33 stimulation. As there is increasing evidence that IL-33 exacerbates a variety of inflammatory conditions such as bronchial inflammation, psoriasis, arthritis and ulcerative colitis,[14] an important role for IL-33 in acute pancreatitis pathogenesis deserves active investigation. Although IL-33 and mast cell interactions may contribute to disease pathogenesis, our findings suggest that IL-33 and acinar cell interactions may have a distinctive role in the early phase of the development of acute pancreatic inflammation that is independent of mast cell degranulation.

The 2005 discovery of IL-33 as a new member of the IL-1 superfamily of cytokines has stimulated considerable scientific interest about its potential role in the progression of several inflammatory conditions.[4], [13], [14] In 2010, four independent groups discovered the association between IL-33 expression and human ulcerative colitis.[6], [16]–[18] However, investigations into the role and expression of IL-33 in acute pancreatitis are limited.[20] Ouziel et. al. reported in 2012 that the plasma of patients with acute pancreatitis shows an early increase in IL-33 receptor ST2 levels that also correlates with disease severity in these patients.[20] Their report also showed increased IL-33 concentration in the *serum* of mice with acute pancreatitis induced with a choline-deficient ethionine-supplemented (CDE) diet, and immunohistochemical staining for IL-33 in the *normal* mouse pancreas showed that the acinar cell constitutively expresses IL-33.[20] Additionally, there is a report confirming that IL-33 is expressed in normal human pancreatic acinar cells and ductal cells and that the ST2 receptor is expressed in human pancreatic acinar, ductal and endothelial cells.[19] The same report also indicates that IL-33 is expressed in human pancreatic stellate cells in chronic pancreatitis and pancreatic cancer.[19] A new finding in our present report is the increased IL-33 expression in the *pancreas* in acute pancreatitis. In the investigation of the early stages of disease pathogenesis, it is important to differentiate increases in pancreatic versus circulating IL-33 levels as the latter can also originate from vascular endothelial cells stimulated by other inflammatory mediators involved in systemic inflammation (e.g., TNF-α released from the pancreas).

IL-33 is mainly expressed by epithelial cells, endothelial cells, fibroblasts, mast cells and macrophages[4] and its expression in other cell types is continually being discovered.[8]–[15], [19], [20] Increased IL-33 protein concentration in the pancreas in acute pancreatitis can be attributed to a variety of sources other than acinar cells, such as pancreatic stromal cells, vascular endothelial cells and ductal epithelial cells.[19], [20] Although constitutive IL-33 expression by acinar cells has been reported, previous studies have not investigated acinar cell-derived IL-33 release in response to agonist stimulation.[19], [20] Here we demonstrate that isolated acinar cells treated with TNF-α increase IL-33 release into the medium. As TNF-α is a key initiator of pancreatic inflammation,[42]–[44] and IL-33 is capable of propagating and maintaining a long term inflammatory state in many organs,[14], [15] the release of IL-33 by acinar cells in response to TNF-α is of importance in the mechanism of disease pathogenesis. TNF-α and IL-1β are prominent inducers of IL-33 expression via ERK activation[6] and our results show that TNF-α-stimulated acinar cell IL-33 release is regulated by the ERK MAP kinase pathway. IL-33 secretion promotes adhesion of leukocytes to endothelial cells.[45] IL-33 activates immune cells especially Th2-cells, mast cells, macrophages, basophils and eosinophils, and is associated with MAP kinase activation in these cells, especially ERK activation.[4], [21], [22] IL-33-induced mast cell activation results in the release of TNF-α, IL-1β, IL-6, CCL2/MCP-1, and prostaglandin D2 (PGD2).[21]

We have previously shown ERK activation in the pancreas and lung in ligation-induced acute pancreatitis in mice.[3] Here we have shown that IL-33 increases ERK activation and CXCL2/MIP-2α, CCL2/MCP-1 and IL-6 production in mouse acinar cells and that specific inhibition of ERK with DN.ERK expression inhibits IL-33-induced cytokine production. Taken together, these studies show that mouse pancreatic acinar cells respond to IL-33 stimulation with increased cytokine production and that the ERK MAP kinase pathway is involved in IL-33-induced activation of acinar cell proinflammatory pathways.

In the present study, we also report that the flavonoid luteolin inhibits IL-33-induced IL-6 and CCL2/MCP-1 release from mouse pancreatic acinar cells. Flavonoids are common constituents of certain edible plants and possess anti-inflammatory, anti-oxidant and anti-allergic activities.[46] Flavonoids inhibit the release of pro-inflammatory mediators from mast cells, T-cells and other inflammatory cells and are used in the treatment of various inflammatory and autoimmune diseases.[46] In mice with cerulein-induced acute pancreatitis, pretreatment with the flavonoid quercetin attenuates disease severity.[39] Luteolin is reported to have beneficial effects in lipopolysaccharide-induced acute lung injury in mice.[34] Therefore, flavonoids may potentially have therapeutic benefits in acute pancreatitis.

Our demonstration that the pancreas from rIL-33-injected mice exhibits activation of ERK MAPK and NF-κB subunit p65, and that acinar cells isolated from the same group of mice release increased quantities of proinflammatory cytokines (e.g., IL-6 and CXCL2/MIP-2α), lends credence to our suggestion that the perivascular and interstitial inflammatory changes in the pancreas are due to acinar cell responses to exogenous IL-33 administration. Also, the lack of mast cell degranulation or change in number in response to exogenous IL-33 at the 48-hour time point also supports the acinar cell origin of the acute inflammatory mediators, rather than a mast cell origin, during this period. However, it should be noted that mast cell activation can occur even in the absence of degranulation as Moulin et. al. have shown that IL-33-stimulated mouse bone marrow-derived mast cells release inflammatory mediators *in vitro* although no change in granule number or localization is evident after IL-33 stimulation,[21] and this was confirmed by others *in vitro*.[20], [35] On the other hand, we underline the distinction between mast cell degranulation observed in our studies in the inflamed pancreas after 48 hrs of duct ligation (Fig. 9) that is associated with increased pancreatic IL-33 levels (Fig. 2), and the pancreatic inflammatory response after exogenous IL-33 administration for 48 hrs where the mast cells do not degranulate. Based on this distinction, one could infer that mast cell degranulation in ligation-induced acute pancreatitis may require some other cytokine in addition to IL-33 (e.g., TNF-α, IL-1β), as exogenous IL-33 administration alone is not associated with mast cell degranulation. Another possibility is that substance P, which is also elevated in the pancreas after duct ligation, synergizes with IL-33 to activate mast cells.[12] Whether the pancreatic mast cells are activated or not after exogenous IL-33 administration, even in the absence of degranulation, would require further investigation. For now, the preponderance of evidence indicates that the acinar cells are the predominant source of the cytokines produced by the pancreas that lead to acute pancreatic inflammation in rIL-33-injected mice. This interpretation follows our consideration that if the cytokines were produced by the effect of IL-33 on vascular endothelial cells, epithelial cells, fibroblasts, neutrophils, macrophages or mast cells — rather than acinar cells — then the jejunum of rIL-33-injected mice should also manifest some inflammatory infiltration, as seen in the pancreas, but this was not the case in our study. In summary, we have found that exogenous IL-33 results in ERK MAPK activation (without p38 or JNK activation), NF-κB subunit p65 activation, proinflammatory mediator production, and acute inflammation in the mouse pancreas *in vivo*, supporting our hypothesis that IL-33 exacerbates acute pancreatic inflammation.

Interactions between IL-33 and mast cells in relation to either maintenance of homeostasis or disordered immune regulation leading to inflammation are relatively recent novel discoveries.[4], [21], [23]–[25] Mast cell activation in various experimental models of acute pancreatitis is well established.[26], [36], [37] Yonetci et. al. showed that the mast cell stabilizing agent ketotifen attenuates cerulein-induced acute pancreatitis in rats.[37] Zhao et. al. observed that the mast cell stabilizer cromolyn prevented pulmonary endothelial barrier dysfunction in rats with acute pancreatitis induced by intraductal infusion of taurocholate,[36] while Dib et. al. used the same model and observed beneficial effects in the pancreas and colon.[38] Ouziel et. al. showed pancreatic mast cell activation in CDE diet-induced acute pancreatitis in the interlobular and peripancreatic areas; they also presented evidence supporting greater activation of mast cells in ST2-deficient mice that have more severe pancreatitis, once again suggesting a protective role for the IL-33 receptor ST2 in disease pathogenesis.[20] When activated, mast cells release a variety of inflammatory mediators including histamine, TNF-α, CCL2/MCP-1, PGD2, platelet activating factor and IL-33.[4], [21], [40] Therefore, our finding in the present report that mast cell degranulation occurs in the pancreas and lung in ligation-induced acute pancreatitis in mice and rats is consistent with the body of evidence that mast cells may play a role in disease pathogenesis. Increased circulating histamine levels originating from activated pancreatic mast cells are implicated in the development of acute lung injury in acute pancreatitis.[36] Histamine causes vasodilatation and plasma extravasation, and increased circulating histamine worsens distant organ injury.[36], [40] Substance P, secreted by neural cells and pancreatic acinar cells,[41] is increased in the pancreas in cerulein-induced acute pancreatitis.[47] Elevated substance P levels in the pancreas in experimental models of acute pancreatitis is of significance as substance P increases mouse pancreatic acinar cell release of CXCL2/MIP-2α and CCL2/MCP-1[48], [49] and worsens leaky capillaries in acute inflammation.[50] Interestingly, Theoharides et. al. have shown that IL-33 augments substance P-induced human mast cell secretion of vascular endothelial growth factor (VEGF), indicating a synergism between IL-33 and substance P on mast cell activation.[12] Increased pancreatic levels of substance P, circulating levels of histamine, and mast cell degranulation in the pancreas and lung in ligation-induced acute pancreatitis in mice and rats is supportive evidence implicating a role for mast cells in disease pathogenesis in this experimental model.

In previous studies, we have characterized in detail various aspects of the new experimental model of ligation-induced acute pancreatitis in mice developed in our laboratory.[2], [3], [27] We demonstrated a near-100% mortality in this model, with 75% of mice dying between day 2 and day 4, and median survival of 3 days.[3] Activation of ERK was seen in the pancreas, lung, liver and kidney. Pancreatic morphological changes included edema, vascular congestion, WBC infiltration, occasional hemorrhage and acinar necrosis, and late evidence of apoptosis; the composition of infiltrating WBCs was immunohistochemically identified as mainly neutrophils (MPO stain) and some macrophages (anti-mouse F4/80 antibody).[27] Morphological evidence of acute lung injury included thickening, vascular congestion and neutrophil infiltration of alveolar septa, while macrophages were not seen even with F4/80 IHC.[27] The liver showed hepatocellular necrosis and neutrophil infiltration while the kidney showed tubular injury but without WBC infiltration.[3] Functional evidence of multiorgan dysfunction included reduced pulmonary compliance,[2] elevated serum creatinine and aspartate aminotransferase, hypotension and progressive bradycardia.[3] The model resembles a recently identified clinical subgroup of patients called “early severe acute pancreatitis” where the patients present with organ failure at admission and have a high mortality rate.[51]–[54] By extending several of our observations in the mouse model to the non-fatal rat model in the present study, we have shown parallel outcomes of duct ligation-induced acute pancreatitis in two species which indicates that our findings are not species specific and that there is a higher probability of clinical relevance to our work.

In conventional experimental models of acute pancreatitis, TNF-α and IL-1β have been shown to play an important role in the early stages of disease pathogenesis.[42], [43] We have previously shown that TNF-α and IL-1β concentrations are increased in the plasma and bronchoalveolar lavage fluid in ligation-induced acute pancreatitis in mice.[3] Here, we extended our observations to confirm increased expression of TNF-α, IL-1β, IL-6, CXCL2/MIP-2α and CCL2/MCP-1 in the pancreas of pancreatitic mice; in addition, we have also shown that IL-6, CXCL2/MIP-2α and CCL2/MCP-1 are increased in the lungs of pancreatitic mice (Fig. 10). In our *in vitro* studies in the present study, we have shown that TNF-α stimulates IL-33 production by acinar cells (Fig. 4) and that IL-33 stimulates acinar cell production of IL-6, CXCL2/MIP-2α and CCL2/MCP-1 (Fig. 5). Taken together, our findings are consistent with our hypothesis that IL-33 plays a role during the early stages of disease pathogenesis. The potential mechanism may involve TNF-α production in the exocrine pancreas followed by TNF-α-induced IL-33 expression by acinar cells, followed by IL-33-induced IL-6 and chemokine (CXCL2/MIP-2α, CCL2/MCP-1) production by acinar cells and by other cells such as mast cells and marcophages.[55] Neutrophil chemotaxis and macrophage activation are known to be stimulated by chemokines such as CXCL2/MIP-2α and CCL2/MCP-1, respectively.[56]–[58] Additionally, IL-33 is known to synergize with chemokines and to act directly via the ST2 receptor to increase neutrophil activation and recruitment.[59]

### Mechanistic Hypothesis

**Figure 12:**
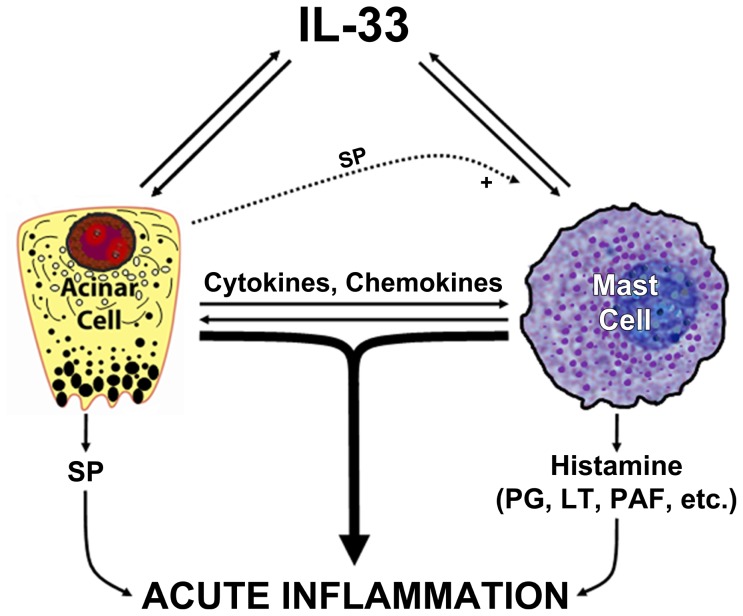
Schematic diagram showing interactions between IL-33, acinar cells and mast cells. Stressed, injured or necrotic acinar cells release IL-33. Mast cells are sensors of cell injury through IL-33 recognition, and activated mast cells release additional IL-33. Acinar cells produce substance P that synergizes with IL-33 to increase mast cell activation. Activated mast cells produce histamine and a large variety of proinflammatory mediators that increase acinar cell stress. In this manner, vicious circles that escalate acute inflammation are generated by the triangular involvement of acinar cells, IL-33 and mast cells. This schema is an intentionally simplified illustration that focuses on certain aspects investigated in the present study, and is only a part of a much more complex environment of proinflammatory and anti-inflammatory signals. SP: substance P, PG: prostaglandins, LT: leukotrienes, PAF: platelet activating factor.

Based on our findings in the present study, we propose a mechanistic hypothesis for the exacerbation of acute pancreatitis in our experimental model. We have shown that acinar cells release and respond to IL-33, while it is already known that mast cells also release and respond to IL-33. Therefore, as illustrated in our schematic diagram (Fig. 12), we propose a triangle of interactions between IL-33, acinar cells and mast cells. Novel interactions between IL-33 and acinar cells have been elucidated for the first time in the present study using both *in vitro* and *in vivo* studies. In addition, we propose that there exist novel interactions between acinar cells and mast cells that have not been elucidated until now and that need to be investigated in future studies.

According to our proposed scheme, acinar cell stress in early stages of acute pancreatitis produces IL-33 and also other cytokines (e.g., TNF-α, IL-1β) that again stimulate acinar cells to release more IL-33. Acinar cell-derived IL-33 activates mast cells. Mast cell-derived IL-33 stimulates acinar cells to further escalate the pro-inflammatory cycle. Stressed acinar cells also secrete substance P that augments the effects of IL-33 on mast cells. Other cytokines produced by acinar cells not only activate mast cells but also promote mast cell degranulation. Mast cell degranulation releases several inflammatory mediators, including histamine that has notable vascular effects promoting pancreatic inflammation, thus increasing acinar cell stress. IL-33-stimulated acinar cells also produce cytokines (e.g., IL-6, CXCL2/MIP-2α and CCL2/MCP-1) that contribute to neutrophil chemotaxis and macrophage activation.[55]–[58] Substance P produced by acinar cells potentially augments effects of IL-33 on mast cells.[12] Therefore, acinar cells, IL-33 and mast cells mutually interact with each other in vicious circles that escalate pancreatic inflammation. The individual and combined effects of these events are probably important for the propagation and maintenance of acute pancreatic inflammation and the consequent systemic inflammatory response syndrome that ultimately leads to mortality in our mouse model of ligation-induced acute pancreatitis.

Additional outcomes of interactions between IL-33, acinar cells and mast cells, involving inflammatory molecules and immune system cells not investigated in the present study, are also possible and have been partly mentioned in our discussion. The scheme that we have presented here is a focused and simplified one that concentrates on aspects investigated in the present study. Future directions of investigation should include studies of the ST2 receptor on acinar cells to better understand the role of IL-33 and its receptor in acute pancreatitis pathogenesis.

### Conclusions

IL-33 stimulates pancreatic acinar cell pro-inflammatory pathways, is induced in stimulated acinar cells and in ligation-induced acute pancreatitis, and exacerbates acute pancreatic inflammation in mice. The acinar cell release of CXCL2/MIP-2α (and potentially other cytokines) in response to IL-33 stimulation, and the increased expression of IL-33 following TNF-α stimulation, involve the ERK MAP kinase signaling pathway. Exogenous IL-33 induces acute pancreatitis in mice, likely by stimulating acinar cell production of inflammatory mediators, by a mechanism that does not involve mast cell degranulation in the early phase (but may or may not involve mast cell activation without degranulation). Mast cell degranulation occurs in the pancreas and lung in pancreatic duct ligation-induced acute pancreatitis in mice and rats, and is associated with increased pancreatic substance P levels and circulating histamine levels. Several cytokines are increased in the pancreas and lung of mice with ligation-induced acute pancreatitis. IL-33 stimulation activates ERK MAP kinase in human pancreatic tissue.

## References

[1] Ranson J, Rifkind K, Roses DF, Fink S, Eng K, et al (1974) Prognostic Signs And The Role Of Operative Management In Acute Pancreatitis. Surgery 139: 69–81. 4834279

[2] Samuel I, Yuan Z, Meyerholz DK, Twait EC, Williard DE, et al (2010) Rapid Communication: A novel model of severe gallstone pancreatitis - Murine pancreatic duct ligation results in systemic inflammation and substantial mortality. Pancreatology 10: 536–544. 20975317 10.1159/000320776 PMC2992635

[3] Yuan Z, Meyerholz DK, Twait EC, Kempuraj D, Williard DE, et al (2011) Systemic Inflammation with Multiorgan Dysfunction Is the Cause of Death in Murine Ligation-Induced Acute Pancreatitis. J Gastrointest Surg 15: 1670–1678 10.1007/s11605-011-1643-2 [doi]. 21800226 10.1007/s11605-011-1643-2

[4] Schmitz J, Owyang A, Oldham E, Song Y, Murphy E, et al (2005) IL-33, an interleukin-1-like cytokine that signals via the IL-1 receptor-related protein ST2 and induces T helper type 2-associated cytokines. Immunity 23: 479–490 S1074-7613(05)00311-0 [pii];10.1016/j.immuni.2005.09.015 [doi]. 16286016 10.1016/j.immuni.2005.09.015

[5] Zhao W, Hu Z (2010) The enigmatic processing and secretion of interleukin-33. Cell Mol Immunol 7: 260–262 cmi20103 [pii];10.1038/cmi.2010.3 [doi]. 20305685 10.1038/cmi.2010.3 PMC4003232

[6] Kobori A, Yagi Y, Imaeda H, Ban H, Bamba S, et al (2010) Interleukin-33 expression is specifically enhanced in inflamed mucosa of ulcerative colitis. J Gastroenterol 45: 999–1007 10.1007/s00535-010-0245-1 [doi]. 20405148 10.1007/s00535-010-0245-1

[7] Benoist C, Mathis D (2002) Mast cells in autoimmune disease. Nature 420: 875–878 10.1038/nature01324 [doi];nature01324 [pii]. 12490961 10.1038/nature01324

[8] Liu X, Li M, Wu Y, Zhou Y, Zeng L, et al (2009) Anti-IL-33 antibody treatment inhibits airway inflammation in a murine model of allergic asthma. Biochem Biophys Res Commun 386: 181–185 S0006-291X(09)01131-0 [pii];10.1016/j.bbrc.2009.06.008 [doi]. 19508862 10.1016/j.bbrc.2009.06.008

[9] Zhiguang X, Wei C, Steven R, Wei D, Wei Z, et al (2010) Over-expression of IL-33 leads to spontaneous pulmonary inflammation in mIL-33 transgenic mice. Immunol Lett 131: 159–165 S0165-2478(10)00122-7 [pii];10.1016/j.imlet.2010.04.005 [doi]. 20412815 10.1016/j.imlet.2010.04.005

[10] Palmer G, Talabot-Ayer D, Lamacchia C, Toy D, Seemayer CA, et al (2009) Inhibition of interleukin-33 signaling attenuates the severity of experimental arthritis. Arthritis Rheum 60: 738–749 10.1002/art.24305 [doi]. 19248109 10.1002/art.24305

[11] Hueber AJ, Alves-Filho JC, Asquith DL, Michels C, Millar NL, et al (2011) IL-33 induces skin inflammation with mast cell and neutrophil activation. Eur J Immunol 41: 2229–2237 10.1002/eji.201041360 [doi]. 21674479 10.1002/eji.201041360

[12] Theoharides TC, Zhang B, Kempuraj D, Tagen M, Vasiadi M, et al (2010) IL-33 augments substance P-induced VEGF secretion from human mast cells and is increased in psoriatic skin. Proc Natl Acad Sci U S A 107: 4448–4453 1000803107 [pii];10.1073/pnas.1000803107 [doi]. 20160089 10.1073/pnas.1000803107 PMC2840132

[13] Pastorelli L, De SC, Cominelli MA, Vecchi M, Pizarro TT (2011) Novel cytokine signaling pathways in inflammatory bowel disease: insight into the dichotomous functions of IL-33 during chronic intestinal inflammation. Therap Adv Gastroenterol 4: 311–323 10.1177/1756283X11410770 [doi];10.1177_1756283X11410770 [pii]. 10.1177/1756283X11410770 PMC3165208 21922030

[14] Miller AM (2011) Role of IL-33 in inflammation and disease. J Inflamm (Lond) 8: 22 1476-9255-8-22 [pii];10.1186/1476-9255-8-22 [doi]. 21871091 10.1186/1476-9255-8-22 PMC3175149

[15] Liew FY, Pitman NI, McInnes IB (2010) Disease-associated functions of IL-33: the new kid in the IL-1 family. Nat Rev Immunol 10: 103–110 nri2692 [pii];10.1038/nri2692 [doi]. 20081870 10.1038/nri2692

[16] Pastorelli L, Garg RR, Hoang SB, Spina L, Mattioli B, et al (2010) Epithelial-derived IL-33 and its receptor ST2 are dysregulated in ulcerative colitis and in experimental Th1/Th2 driven enteritis. Proc Natl Acad Sci U S A 107: 8017–8022 0912678107 [pii];10.1073/pnas.0912678107 [doi]. 20385815 10.1073/pnas.0912678107 PMC2867895

[17] Beltran CJ, Nunez LE, Diaz-Jimenez D, Farfan N, Candia E, et al (2010) Characterization of the novel ST2/IL-33 system in patients with inflammatory bowel disease. Inflamm Bowel Dis 16: 1097–1107 10.1002/ibd.21175 [doi]. 20014018 10.1002/ibd.21175

[18] Seidelin JB, Bjerrum JT, Coskun M, Widjaya B, Vainer B, et al (2010) IL-33 is upregulated in colonocytes of ulcerative colitis. Immunol Lett 128: 80–85 S0165-2478(09)00270-3 [pii];10.1016/j.imlet.2009.11.001 [doi]. 19913053 10.1016/j.imlet.2009.11.001

[19] Masamune A, Watanabe T, Kikuta K, Satoh K, Kanno A, et al (2010) Nuclear expression of interleukin-33 in pancreatic stellate cells. Am J Physiol Gastrointest Liver Physiol 299: G821–G832 ajpgi.00178.2010 [pii];10.1152/ajpgi.00178.2010 [doi]. 20689058 10.1152/ajpgi.00178.2010

[20] Ouziel R, Gustot T, Moreno C, Arvanitakis M, Degre D, et al (2012) The ST2 Pathway Is Involved in Acute Pancreatitis: A Translational Study in Humans and Mice. Am J Pathol 180 S0002-9440(12)00248-9 [pii];10.1016/j.ajpath.2012.03.009 [doi]. 10.1016/j.ajpath.2012.03.009 PMC3366073 22542450

[21] Moulin D, Donze O, Talabot-Ayer D, Mezin F, Palmer G, et al (2007) Interleukin (IL)-33 induces the release of pro-inflammatory mediators by mast cells. Cytokine 40: 216–225 S1043-4666(07)00428-0 [pii];10.1016/j.cyto.2007.09.013 [doi]. 18023358 10.1016/j.cyto.2007.09.013

[22] Yagami A, Orihara K, Morita H, Futamura K, Hashimoto N, et al (2010) IL-33 mediates inflammatory responses in human lung tissue cells. J Immunol 185: 5743–5750 jimmunol.0903818 [pii];10.4049/jimmunol.0903818 [doi]. 20926795 10.4049/jimmunol.0903818

[23] Silver MR, Margulis A, Wood N, Goldman SJ, Kasaian M, et al (2010) IL-33 synergizes with IgE-dependent and IgE-independent agents to promote mast cell and basophil activation. Inflamm Res 59: 207–218 10.1007/s00011-009-0088-5 [doi]. 19763788 10.1007/s00011-009-0088-5

[24] Enoksson M, Lyberg K, Moller-Westerberg C, Fallon PG, Nilsson G, et al (2011) Mast cells as sensors of cell injury through IL-33 recognition. J Immunol 186: 2523–2528 jimmunol.1003383 [pii];10.4049/jimmunol.1003383 [doi]. 21239713 10.4049/jimmunol.1003383

[25] Lunderius-Andersson C, Enoksson M, Nilsson G (2012) Mast Cells Respond to Cell Injury through the Recognition of IL-33. Front Immunol 3: 82 10.3389/fimmu.2012.00082 [doi]. 22566963 10.3389/fimmu.2012.00082 PMC3342375

[26] Lopez-Font I, Gea-Sorli S, de-Madaria E, Gutierrez LM, Perez-Mateo M, et al (2010) Pancreatic and pulmonary mast cells activation during experimental acute pancreatitis. World J Gastroenterol 16: 3411–3417. 20632444 10.3748/wjg.v16.i27.3411 PMC2904888

[27] Meyerholz DK, Williard DE, Grittmann AM, Samuel I (2008) Murine pancreatic duct ligation induces stress kinase activation, acute pancreatitis, and acute lung injury. Am J Surg 196: 675–682. 18789417 10.1016/j.amjsurg.2008.07.009 PMC13375276

[28] Williard DE, Twait E, Yuan Z, Carter AB, Samuel I (2010) Nuclear factor kappa B-dependent gene transcription in cholecystokinin- and tumor necrosis factor-alpha-stimulated isolated acinar cells is regulated by p38 mitogen-activated protein kinase. Am J Surg 200: 283–290 S0002-9610(10)00070-X [pii];10.1016/j.amjsurg.2009.12.004 [doi]. 20413104 10.1016/j.amjsurg.2009.12.004 PMC2910146

[29] Bi Y, Page SL, Williams JA (2005) Rho and Rac promote acinar morphological changes, actin reorganization, and amylase secretion. Am J Physiol Gastrointest Liver Physiol 289: G561–G570. 15920016 10.1152/ajpgi.00508.2004

[30] Lefrancais E, Roga S, Gautier V, Gonzalez-de-Peredo A, Monsarrat B, et al (2012) IL-33 is processed into mature bioactive forms by neutrophil elastase and cathepsin G. Proc Natl Acad Sci U S A 109: 1673–1678 1115884109 [pii];10.1073/pnas.1115884109 [doi]. 22307629 10.1073/pnas.1115884109 PMC3277172

[31] Meyerholz DK, Samuel I (2007) Morphologic characterization of early ligation-induced acute pancreatitis in rats. Am J Surg 194: 652–658. 17936429 10.1016/j.amjsurg.2007.07.014 PMC2128702

[32] Churg A, Richter WR (1971) Early changes in the exocrine pancreas of the dog and rat after ligation of the pancreatic duct. A light and electron microscopic study. Am J Pathol 63: 521–546. 5581235 PMC2047483

[33] Hsu CL, Neilsen CV, Bryce PJ (2010) IL-33 is produced by mast cells and regulates IgE-dependent inflammation. PLoS One 5: e11944 10.1371/journal.pone.0011944 [doi]. 20689814 10.1371/journal.pone.0011944 PMC2914748

[34] Lee JP, Li YC, Chen HY, Lin RH, Huang SS, et al (2010) Protective effects of luteolin against lipopolysaccharide-induced acute lung injury involves inhibition of MEK/ERK and PI3K/Akt pathways in neutrophils. Acta Pharmacol Sin 31: 831–838 aps201062 [pii];10.1038/aps.2010.62 [doi]. 20562902 10.1038/aps.2010.62 PMC4007725

[35] Kandere-Grzybowska K, Letourneau R, Kempuraj D, Donelan J, Poplawski S, et al (2003) IL-1 induces vesicular secretion of IL-6 without degranulation from human mast cells. J Immunol 171: 4830–4836. 14568962 10.4049/jimmunol.171.9.4830

[36] Zhao X, Dib M, Wang X, Widegren B, Andersson R (2005) Influence of mast cells on the expression of adhesion molecules on circulating and migrating leukocytes in acute pancreatitis-associated lung injury. Lung 183: 253–264 . 16211461 10.1007/s00408-004-2538-8

[37] Yonetci N, Oruc N, Ozutemiz AO, Celik HA, Yuce G (2001) Effects of mast-cell stabilization in cerulein-induced acute pancreatitis in rats. Int J Pancreatol 29: 163–171 10.1385/IJGC:29:3:163 [doi]. 12067220 10.1385/IJGC:29:3:163

[38] Dib M, Zhao X, Wang XD, Andersson R (2002) Role of mast cells in the development of pancreatitis-induced multiple organ dysfunction. Br J Surg 89: 172–178 1991 [pii];10.1046/j.0007-1323.2001.01991.x [doi]. 11856129 10.1046/j.0007-1323.2001.01991.x

[39] Carvalho KM, Morais TC, de Melo TS, de Castro Brito GA, de Andrade GM, et al (2010) The natural flavonoid quercetin ameliorates cerulein-induced acute pancreatitis in mice. Biol Pharm Bull 33: 1534–1539 JST.JSTAGE/bpb/33.1534 [pii]. 20823570 10.1248/bpb.33.1534

[40] Nathan C (2002) Points of control in inflammation. Nature 420: 846–852 10.1038/nature01320 [doi];nature01320 [pii]. 12490957 10.1038/nature01320

[41] Tamizhselvi R, Moore PK, Bhatia M (2007) Hydrogen sulfide acts as a mediator of inflammation in acute pancreatitis: in vitro studies using isolated mouse pancreatic acinar cells. J Cell Mol Med 11: 315–326 JCMM024 [pii];10.1111/j.1582-4934.2007.00024.x [doi]. 17488480 10.1111/j.1582-4934.2007.00024.x PMC3822830

[42] Norman J (1998) The role of cytokines in the pathogenesis of acute pancreatitis. Am J Surg 175: 76–83. 9445247 10.1016/s0002-9610(97)00240-7

[43] Bhatia M, Brady M, Shokuhi S, Christmas S, Neoptolemos JP, et al (2000) Inflammatory mediators in acute pancreatitis. J Pathol 190: 117–125. 10657008 10.1002/(SICI)1096-9896(200002)190:2<117::AID-PATH494>3.0.CO;2-K

[44] Norman JG, Fink GW, Messina J, Carter G, Franz MG (1996) Timing of tumor necrosis factor antagonism is critical in determining outcome in murine lethal acute pancreatitis. Surgery 120: 515–521. 8784406 10.1016/s0039-6060(96)80072-9

[45] Demyanets S, Konya V, Kastl SP, Kaun C, Rauscher S, et al (2011) Interleukin-33 induces expression of adhesion molecules and inflammatory activation in human endothelial cells and in human atherosclerotic plaques. Arterioscler Thromb Vasc Biol 31: 2080–2089 ATVBAHA.111.231431 [pii];10.1161/ATVBAHA.111.231431 [doi]. 21737781 10.1161/ATVBAHA.111.231431

[46] Lopez-Lazaro M (2009) Distribution and biological activities of the flavonoid luteolin. Mini Rev Med Chem 9: 31–59. 19149659 10.2174/138955709787001712

[47] Bhatia M, Saluja AK, Hofbauer B, Frossard JL, Lee HS, et al (1998) Role of substance P and the neurokinin 1 receptor in acute pancreatitis and pancreatitis-associated lung injury. Proc Natl Acad Sci U S A 95: 4760–4765. 9539812 10.1073/pnas.95.8.4760 PMC22564

[48] Ramnath RD, Sun J, Bhatia M (2008) Role of calcium in substance P-induced chemokine synthesis in mouse pancreatic acinar cells. Br J Pharmacol 154: 1339–1348 bjp2008188 [pii];10.1038/bjp.2008.188 [doi]. 18493246 10.1038/bjp.2008.188 PMC2483386

[49] Ramnath RD, Sun J, Adhikari S, Bhatia M (2007) Effect of mitogen-activated protein kinases on chemokine synthesis induced by substance P in mouse pancreatic acinar cells. J Cell Mol Med 11: 1326–1341. 18205703 10.1111/j.1582-4934.2007.00086.x PMC4401295

[50] Bhatia M (2010) Hydrogen sulfide and substance P in inflammation. Antioxid Redox Signal 12: 1191–1202 10.1089/ars.2009.2927 [doi]. 19803739 10.1089/ars.2009.2927

[51] Johnson CD, bu-Hilal M (2004) Persistent organ failure during the first week as a marker of fatal outcome in acute pancreatitis. Gut 53: 1340–1344. 15306596 10.1136/gut.2004.039883 PMC1774183

[52] Isenmann R, Rau B, Beger HG (2001) Early severe acute pancreatitis: characteristics of a new subgroup. Pancreas 22: 274–278. 11291929 10.1097/00006676-200104000-00008

[53] McKay CJ, Evans S, Sinclair M, Carter CR, Imrie CW (1999) High early mortality rate from acute pancreatitis in Scotland, 1984–1995. Br J Surg 86: 1302–1305. 10540138 10.1046/j.1365-2168.1999.01246.x

[54] Tenner S, Sica G, Hughes M, Noordhoek E, Feng S, et al (1997) Relationship of necrosis to organ failure in severe acute pancreatitis. Gastroenterology 113: 899–903. 9287982 10.1016/s0016-5085(97)70185-9

[55] Milovanovic M, Volarevic V, Radosavljevic G, Jovanovic I, Pejnovic N, et al (2012) IL-33/ST2 axis in inflammation and immunopathology. Immunol Res 52: 89–99 10.1007/s12026-012-8283-9 [doi]. 22392053 10.1007/s12026-012-8283-9

[56] Kobayashi Y (2008) The role of chemokines in neutrophil biology. Front Biosci 13: 2400–2407 2853 [pii]. 17981721 10.2741/2853

[57] Deshmane SL, Kremlev S, Amini S, Sawaya BE (2009) Monocyte chemoattractant protein-1 (MCP-1): an overview. J Interferon Cytokine Res 29: 313–326 10.1089/jir.2008.0027 [doi]. 19441883 10.1089/jir.2008.0027 PMC2755091

[58] Yadav A, Saini V, Arora S (2010) MCP-1: chemoattractant with a role beyond immunity: a review. Clin Chim Acta 411: 1570–1579 S0009-8981(10)00465-1 [pii];10.1016/j.cca.2010.07.006 [doi]. 20633546 10.1016/j.cca.2010.07.006

[59] Verri WA Jr, Souto FO, Vieira SM, Almeida SC, Fukada SY, et al (2010) IL-33 induces neutrophil migration in rheumatoid arthritis and is a target of anti-TNF therapy. Ann Rheum Dis 69: 1697–1703 ard.2009.122655 [pii];10.1136/ard.2009.122655 [doi]. 20472598 10.1136/ard.2009.122655

